# Associations between cortical thickness and reasoning differ by socioeconomic status in development

**DOI:** 10.1016/j.dcn.2019.100641

**Published:** 2019-03-23

**Authors:** Julia A. Leonard, Rachel R. Romeo, Anne T. Park, Megumi E. Takada, Sydney T. Robinson, Hannah Grotzinger, Briana S. Last, Amy S. Finn, John D.E. Gabrieli, Allyson P. Mackey

**Affiliations:** a Department of Brain and Cognitive Sciences and McGovern Institute for Brain Research, Massachusetts Institute of Technology, 43 Vassar St Room 46-4033, Cambridge, MA, 02139, USA; b Division of Medical Sciences, Harvard University, 260 Longwood Ave, T-MEC 435, Boston, MA, 02155, USA; c Department of Psychology, University of Pennsylvania, Levin Building 425 S. University Ave, Room 354, Philadelphia, PA, 19104, USA; d Department of Psychology, University of Toronto, 100 St. George St, Sidney Smith Hall, Toronto, ON, M5S 3G3, Canada

**Keywords:** Brain development, Child development, Socioeconomic status, Reasoning, MRI, early childhood

## Abstract

Although lower socioeconomic status (SES) is generally negatively associated with performance on cognitive assessments, some children from lower-SES backgrounds perform as well as their peers from higher-SES backgrounds. Yet little research has examined whether the neural correlates of individual differences in cognition vary by SES. The current study explored whether relationships between cortical structure and fluid reasoning differ by SES in development. Fluid reasoning, a non-verbal component of IQ, is supported by a distributed frontoparietal network, with evidence for a specific role of rostrolateral prefrontal cortex (RLPFC). In a sample of 115 4–7-year old children, bilateral thickness of RLPFC differentially related to reasoning by SES: thicker bilateral RLPFC positively correlated with reasoning ability in children from lower-SES backgrounds, but not in children from higher-SES backgrounds. Similar results were found in an independent sample of 59 12–16-year old adolescents. Furthermore, young children from lower-SES backgrounds with strong reasoning skills were the only group to show a positive relationship between RLPFC thickness and age. In sum, we found that relationships between cortical thickness and cognition differ by SES during development.

## Highlights

• Thickness of RLPFC positively relates to reasoning in 4–7-year olds from lower, but not higher, SES backgrounds.
• The positive relationship between RLPFC thickness and reasoning was also present in a sample of 12–16-year olds from lower-SES backgrounds.
• Young children with strong reasoning from lower-SES backgrounds uniquely showed positive relationships between RLPFC thickness and age.
• This work suggests that the neural structure that supports reasoning varies by SES during development.

## 1 Introduction

The gap in academic achievement between children from higher- and lower-socioeconomic status (SES) backgrounds is present early in childhood, even before children enter school (Duncan and Brooks-Gunn, 1997). However, some children from lower-SES backgrounds have strong cognitive skills and excel in school, performing just as well as or better than their peers from higher-SES backgrounds (Borman and Overman, 2004; Masten, 2001; Waxman et al., 2003; Werner and Smith, 2001). Understanding neural correlates of cognition in young children from lower-SES backgrounds could help elucidate how the environment influences brain-behavior relationships, and, ultimately, set targets for early interventions. Research on brain-behavior correlations has often implicitly assumed that such relationships are generalizable across children from all backgrounds, rather than testing whether such correlations differ by SES. However, an evolutionary-developmental perspective suggests that variation in early experience may lead to specific neural and cognitive adaptations to fit these environments (Ellis et al., 2017). By this view, optimal brain-behavior development may differ across SES, with neural correlates of strong cognitive skills differing by early life environment. Here, we aimed to test whether cognition in children from lower-SES environments is related to different patterns of neural structure than cognition in children from higher-SES backgrounds.

Many studies linking neural structure to cognition have focused on full-scale IQ (FSIQ). FSIQ is composed of two components: verbal IQ and performance IQ. Verbal IQ relies on crystallized knowledge, such as vocabulary. Performance IQ includes fluid reasoning, visuospatial skills, working memory, and processing speed, and is less dependent on prior knowledge (Cattell, 1943). In adults, higher FSIQ is related to thicker cortex in rostrolateral prefrontal cortex (RLPFC), and in posterior temporal and visual areas (Choi et al., 2008; Narr et al., 2007). Two developmental studies have found results that are consistent with this adult pattern (9–24-year olds in Menary et al., 2013; 6–18-year olds in Karama et al., 2009). However, two other developmental studies have shown the reverse pattern, with thinner cortex being associated with superior performance. Thinner parietal cortices related to better performance on verbal and non-verbal cognitive measures between the ages of 12 and 14 years (Squeglia et al., 2013), and globally thinner cortex related to higher FSIQ between the ages of 10 and 20 (Schnack et al., 2015). Inconsistencies in associations between cortical thickness and IQ could be due to factors including methodological differences, data quality, and demographics of the sample, such as age and SES.

Longitudinal studies have shown that the trajectory of cortical development, rather than thickness at a single time point, best relates to FSIQ (Schnack et al., 2015; Shaw et al., 2006). Individuals with higher FSIQs show greater changes in cortical thickness across the lifespan: greater cortical thickening from age 7 until age 12 and then greater thinning until age 25. In contrast, individuals with lower FSIQs show consistent thinning throughout the lifespan. Thus, relationships between cortical thickness and cognition might change throughout development. However, it is currently unknown how cortical thickness relates to cognition before age 7 even at a single time point, and how this relationship varies by SES.

Lower-SES has been related to brain structure (reduced gray matter volume, surface area, and cortical thickness), and in turn, lower cognitive skills (Farah, 2017). The relationship between cortical thickness and SES varies by age: thickness-SES relationships are small in early childhood, grow in adolescence, then narrow again in adulthood (Piccolo et al., 2016). However, the diversity of brain structure and cognitive performance *within* lower-SES students is poorly understood. One study found that SES moderates relationships between cortical thickness and cognition: in children with thicker cortex, SES correlated positively with executive function, and in children with thinner cortex, SES correlated positively with language abilities (Brito, Piccolo, Noble, & Pediatric Imaging, Neurocognition, and Genetics Study, 2017). However, this study was conducted in a large age range (3–18), leaving open the question of whether SES differences in brain-behavior relationships change with age.

In the current study, we focused on fluid reasoning, a component of performance IQ that is highly correlated with academic performance (Ferrer and McArdle, 2004; Floyd et al., 2003; Fuchs et al., 2006) and differs by SES (Finn et al., 2016; Platt et al., 2018). Fluid reasoning, commonly measured by matrix reasoning tests (Raven, 1965), has been well-characterized from a neural perspective: it relies on a distributed frontoparietal network, with RLPFC playing a specific role in relational reasoning (Bunge et al., 2009; Christoff et al., 2001; Crone et al., 2009; Dumontheil, 2014; Wendelken et al., 2012, 2016; Wendelken et al., 2011). In an exploratory analysis, we asked whether relationships between fluid reasoning and cortical structure differed by SES in early childhood. This analysis led to specific predictions about where and how cortical thickness relates to reasoning differently by SES, which we then tested in an independent sample of adolescents. Given evidence that the rate of thinning, rather than static thickness, relates to cognition, we also explored whether cross-sectional associations between cortical thickness and age differed by SES and reasoning ability. To our knowledge, this is the first study to examine whether relationships between brain structure and reasoning differ by SES during development.

## 2 Methods

This study was approved by the Committee on the Use of Humans as Experimental Subjects at the Massachusetts Institute of Technology (MIT). Participants under the age of eight provided verbal assent, and participants eight and older provided written assent. All parents provided written consent.

### 2.1 Participants

#### 2.1.1 Early childhood sample (ECS)

Children were recruited from the Greater Boston Area as part of two larger studies: one on executive function development and the other exploring a parenting intervention (only data prior to the intervention were included). Recruitment for the executive function study occurred through postings on parent forums, magazine ads, community family events, and Head Start programs. Recruitment for the intervention study occurred mainly through charter schools. Analyses of functional imaging data collected as part of these studies have been published previously (Park et al., 2018; Romeo et al., 2018a,b). Across both studies, a total of 130 children underwent structural imaging. In screening interviews for both studies, parents reported whether their children were born prematurely or had medically diagnosed neurological or psychiatric disorders. Children were excluded from analysis if they had a medical diagnosis (n = 8), excessive motion artifacts (n = 6; see Structural Imaging Analysis), or missing information on maternal education (*n* = 1). The final sample consisted of 115 children (53 female, mean age (SD) = 5.85(0.96), age range: 4–7 years, see Table S1 for demographics).

#### 2.1.2 Adolescent sample (AS)

Adolescents were recruited from the Greater Boston Area as part of a larger study exploring SES, brain development, and educational outcomes. Analyses of subsets of this dataset have been published previously (Finn et al., 2016; Leonard et al., 2015; Mackey et al., 2015). Participants were excluded in the current analysis if they did not complete a structural scan (*n* = 7), had missing data on matrix reasoning (*n* = 2), or were missing data on maternal education (*n* = 23). The final sample consisted of 59 adolescents (28 female, mean age (SD) = 14.44(0.55), age range: 12–16 years, see Table S1 for demographics).

### 2.2 Matrix reasoning

All children in the early childhood sample completed the Matrix Reasoning subscale of the Wechsler Preschool and Primary Scale of Intelligence (WPPSI-IV; Wechsler, 2012). The Matrix Reasoning subtest required children to pick the picture that completed the missing piece of a colored puzzle. Problems started out easy and progressively got harder. These problems required children to integrate information about semantic relationships, shape, pattern, and orientation. Children in the executive function study completed testing in a quiet room, while most of the children in the intervention study completed testing in school settings. Matrix reasoning scores did not differ by study (standard score: t(112)=1.24, p=.219). One child was missing an age-adjusted standard score due to being older than the WPPSI age norms (limit is 7.58 years, child was 7.92 years). Matrix reasoning raw scores were used in brain imaging analyses, which control for age.

The adolescent sample completed the Test of Nonverbal Intelligence (TONI, Version B, Brown et al., 2010) which measures matrix reasoning ability. Subjects chose which picture completed the missing piece of a puzzle. Puzzles progressively got harder, requiring integration of shape, pattern, and orientation. Unlike the WPPSI matrix reasoning measure, images in the TONI are black and white line drawings. Raw scores were used in brain analyses, which control for age.

### 2.3 Socioeconomic status

SES was operationalized as the highest level of maternal education in years, a measure that is more stable than income and a better index of children’s cognitive environments (Davis-Kean, 2005; ECS: M(SD) = 15.47 (2.91); AS: M(SD) = 15.42 (3.24)). For some analyses, children were split into two groups: children whose mothers did not complete college, referred to throughout as the Lower-SES group (ECS: *n* = 52, 24 F; AS: *n* = 25, 12 F), and children whose mothers did complete college, referred to as the Higher-SES group (ECS: *n* = 63, 29 F; AS: *n* = 34, 16 F). These groups did not differ in age or gender (ECS age: t(113)=-0.16, p=.877; Gender: $\chi^{2}(1,n=115)=0.03$, p= .863; AS age:  t(57)=-1.03, p=.307; Gender: $\chi^{2}(1,n=59)=0.04$, p=.841).

We also ran analyses in the early childhood sample with income as our SES measure to determine whether results were limited to maternal education as the measure of SES. A continuous measure of income was not available in the full adolescent sample. Income and maternal education were highly correlated (t(113)=9.05, p<.001). For some analyses, children were split into two groups based on the median household income in the greater Boston area in 2016, which was approximately $80 K (Lower income group *n* = 58, 23 F; Higher income group *n* = 57, 30 F). These groups did not differ in age or gender (ECS age: t(113)=1.47, p=.145; Gender: $\chi^{2}(1,n=115)=1.46$, p=.227).

The higher-SES group reported the following racial and ethnic identities: ECS 10% African American, 1% Asian, 67% White, 14% multiple races, 8% did not report race; 84% not Hispanic or Latino, 16% Hispanic or Latino, 0% did not report ethnicity; and AS 9% African American, 12% Asian, 47% White, 3% Native Hawaiian or Pacific Islander, 23% multiple races, 6% did not report race; 85% not Hispanic or Latino, 15% Hispanic or Latino, 0% did not report ethnicity. The lower-SES group reported the following racial and ethnic identities: ECS 38% African American, 0% Asian, 15% White, 12% multiple races, 35% did not report race; 60% not Hispanic or Latino, 35% Hispanic or Latino, 5% did not report ethnicity; AS 40% African American, 4% Asian, 12% White, 0% Native Hawaiian or Pacific Islander, 16% multiple races, 28% did not report race; 52% not Hispanic or Latino, 44% Hispanic or Latino, 4% did not report ethnicity. This data mirrors the demographic distribution in the United States, with the lower-SES group containing a larger percentage of ethnic and racial minorities than the higher-SES group. Analyses controlling for race and ethnicity are reported in the Supplemental Material.

### 2.4 Neuroimaging data acquisition

Data were acquired at the Athinoula A. Martinos Imaging Center at MIT. Before the MRI, participants acclimated to the scanning environment and practiced staying still by undergoing a ‘mock scan’. Scanning was performed using a Siemens MAGNETOM Trio Tim 3 T MRI scanner with a 32-channel coil. A whole-brain, high-resolution, T1-weighted multi-echo structural scan (MPRAGE) was collected (ECS acquisition parameters: TR = 2530 ms, TE = 1.64 ms/3.5 ms/5.36 ms/7.22 ms, flip angle = 7°, voxel size = 1 mm isotropic, matrix size = 192 × 192, 176 sagittal slices, FOV = 192 mm; AS: TR = 2530 ms; TE = 1.64 ms/3.44 ms/5.24 ms/7.04 ms; flip angle = 7°; resolution = 1 mm isotropic). This sequence was optimized for participants with high motion (Tisdall et al., 2012). This sequence included the volumetric navigators (vNavs) prospective motion correction system, which tracked the subject's head motion and corrected the imaging coordinates to follow the subject's motion in real time (Tisdall et al., 2012). This method has been shown, when using a very similar scanning protocol, to significantly reduce motion-induced biases in cortical thickness measures (Tisdall et al., 2016).

### 2.5 Structural imaging analyses

Two trained coders who were blind to participant information rated structural images for quality on a scale of 1 (highest quality) to 4 (lowest quality) based on a visual guide of artifacts associated with motion (see Fig. S1). In the early childhood sample, ratings were Z-scored within study due to slightly different methods across studies. In the executive function study, ratings were averaged unless they differed by more than one point, in which case, a third rater made a final decision (M(SD) = 2.55 (0.82)). In the intervention study, two coders separately rated image quality and discussed any discrepancies to come up with a final rating (M(SD) = 2.05 (0.58)). The methodology in the adolescent sample followed that in the executive function study. Ratings did not significantly relate to maternal education (ECS: r(113)=.00, p=.971; AS: r(57)=.04, p=.737), matrix reasoning (ECS: b=-0.04, 95% CI [−0.09, 4×10^−3^], *t*(112) = −1.82 , p=.072; AS: b= 2 × 10^−3^, 95% CI [-0.01, 0.02], *t*(56) = 0.37, p=.715; models control for age), or matrix reasoning by maternal education in an interaction (ECS: b=0.01, 95% CI [-0.01, 0.02], t(110)=0.74, p=.459; AS: b= 2 × 10^−3^, 95% CI [- 2 × 10^−3^, 0.01], *t*(54) = 1.03 , p=.305; model controls for age). All models with brain data control for quality rating.

Structural analyses were conducted in FreeSurfer Version 5.3 (Fischl et al., 2002, 2004). Surfaces were edited as needed, and final surfaces were checked by a coder who was blind to participant demographics and cognitive measures. Six children in the early childhood sample were excluded for low image quality resulting in inaccurate surfaces. Each participant’s surface was resampled to a standard brain (fsaverage) and smoothed with a 15-mm full-width half-maximum kernel.

A general linear model was constructed to test for an interaction between maternal education and matrix reasoning on cortical thickness, defined as the distance between the white matter and pial surface at each cortex location (Fischl and Dale, 2000). To better understand interaction effects, we ran whole-brain analyses relating cortical thickness to matrix reasoning within the lower- and higher-SES subgroups (controlling for maternal education so the results would not be driven by within-group SES gradients). Main effects of maternal education, matrix reasoning, and age on cortical thickness were evaluated separately. All analyses controlled for age (except for the main effect analysis of age), gender, study (in the ECS), and image quality.

Whole-brain analyses were cluster-corrected for multiple comparisons using Monte Carlo simulation (cluster-wise *p* < .05, adjusted for both hemispheres; Hagler et al., 2006). A cluster-forming threshold was set to *p* <  .005 (Greve and Fischl, 2017). For full transparency of how effects change across thresholds, results are shown at multiple cluster-forming thresholds in the supplemental materials. For plotting purposes only, we extracted the parameter estimates from the whole brain analyses. All subsequent analyses were run using anatomically defined regions of interest (ROIs) of the left and right rostral middle frontal gyrus (RMFG), based on an automated gyral-based parcellation from FreeSurfer (Desikan et al., 2006). We opted to use an anatomical ROI primarily for statistical analyses to maintain independence, but also because this larger ROI is more likely to capture the specific position of relational integration areas across individuals.

We ran three-way interaction models with age, maternal education, and matrix reasoning on thickness in left and right rostral middle frontal gyrus (RMFG). For post-hoc linear models, we split the data into four groups: first based on SES, and then again within SES groups based on median matrix reasoning scores.

To determine if our results were limited to maternal education as the SES measure, we repeated the above analyses with income as the SES measure. Results were broadly similar across SES measures. Analyses using income as the SES measure are reported in the supplemental materials (Figs. S5–S9, Table S3).

Finally, given the exploratory nature of our analyses, we aimed to extend our results in an independent sample of 59 adolescents. Due to the specific hypotheses generated from analyses of the early childhood sample, and the smaller size of the adolescent sample, we focused our analyses on *a priori* regions of interest identified in the early childhood sample: left and right RMFG (as defined above). In these regions, we tested our hypothesis of positive correlations between thickness and matrix reasoning within the lower-SES group, but not the higher SES-group. We also tested for main effects of age, matrix reasoning, and maternal education, as well as for a 3-way interaction amongst age, maternal education, and matrix reasoning. Finally, as an exploratory analysis to fully characterize the data, we tested whether these results were significant at the whole brain level.

## 3 Results

### 3.1 Early childhood sample

**Fig. 1:**
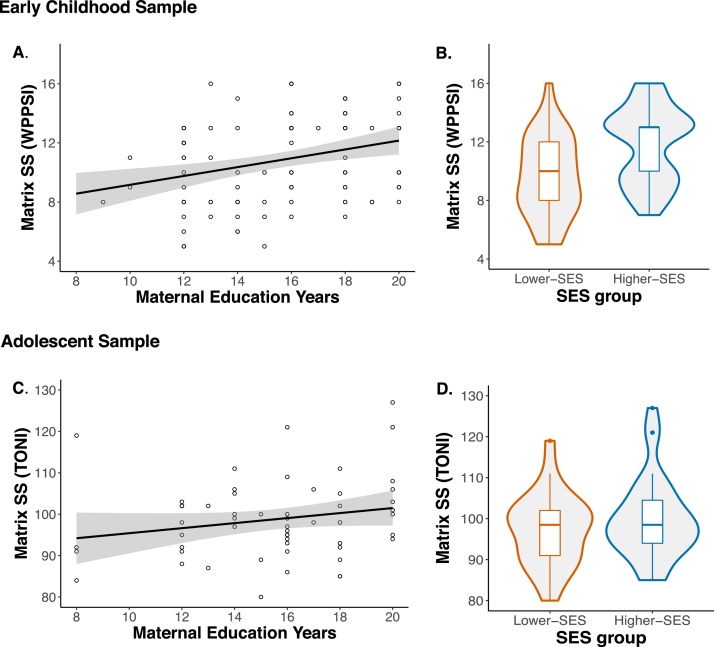
Maternal Education correlated with Matrix Reasoning standard score (SS) in the early childhood sample and adolescent sample. Matrix reasoning was measured using the *Wechsler Preschool and Primary Scale of Intelligence (*WPPSI) matrix reasoning subtest in the early childhood sample and the *Test of Nonverbal Intelligence* (TONI) in the adolescent sample. A. Maternal education in years correlated with matrix reasoning age-normed standard score (Matrix SS) in the early childhood sample (*r*(112) = .31, *p* =  .001). B. Matrix SS differed by SES group in the early childhood sample (Lower-SES: maternal education < 16 years, Higher-SES: maternal education >= 16 years) (*t*(112) = -4.45, *p* <  .001). C. There was a positive trend between maternal education and matrix reasoning standard score in the adolescent sample (*r*(57) = .24, *p* =  .071). D. Matrix SS did not differ by SES group in the adolescent sample (*t*(57) = 1.33, *p* =  .189).

Maternal education positively correlated with matrix reasoning scores ($r\left(112\right)=\;.31$, p=.001; Fig. 1A). Splitting the sample into two groups based on maternal education (< 16 years or > = 16 years) revealed high variability within group (Fig. 1B), and a significant mean difference between groups (t(112)=-4.45, p<.001). Matrix reasoning was positively related to right lateral occipital cortex thickness, and age was negatively related to left pericalcarine thickness (Fig. S2). There were no main effects of maternal education on cortical thickness at the cluster-forming threshold of *p* <  .005 (for more lenient thresholds, see Fig. S2).

**Fig. 2:**
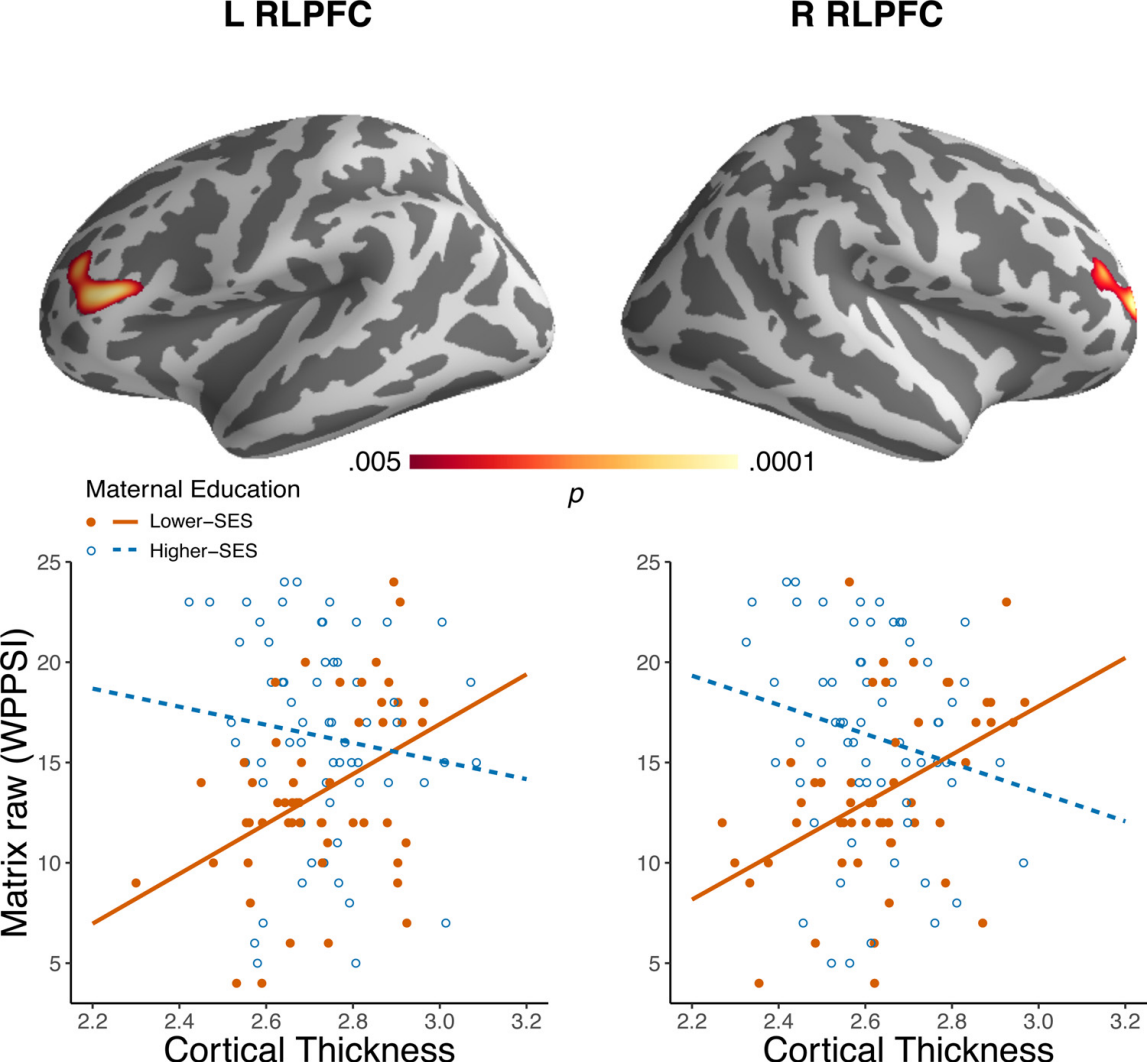
Interaction between matrix reasoning and maternal education on whole brain cortical thickness in the early childhood sample. Age, gender, image quality, and study were included as covariates. Results were cluster corrected for multiple comparisons using Monte Carlo simulations (cluster-forming *p* <  .005, cluster-wise *p* <  .05, adjusted for both hemispheres). Scatterplots show interaction results with extracted parameter estimates (adjusted for covariates) in right and left rostrolateral prefrontal cortex (RLPFC) in mm. Matrix reasoning was measured with the *Wechsler Preschool and Primary Scale of Intelligence (*WPPSI). Maternal education is plotted as a binary variable for display purposes only (Lower-SES group, orange: maternal education < 16 years, Higher-SES group, blue: maternal education > = 16 years). The MNI coordinates for the left cluster peak vertex are -39.4, 28.7, 12.9 (1252 vertices). The MNI coordinates for the right cluster peak vertex are 25.1, 53.5, 9.0 (1115 vertices). The range of cortical thickness estimates in left and right RMFG closely match those presented in the Pediatric Imaging Neurocognition and Genetics Study (PING) dataset (see Piccolo, Brito, & Noble, 2017) (For interpretation of the references to colour in this figure legend, the reader is referred to the web version of this article).

There was a significant interaction between maternal education and matrix reasoning on cortical thickness in RLPFC (Fig. 2; see Fig. S3 for more lenient thresholds). To understand the direction of the interaction, and to confirm that it was not driven by outliers, we plotted parameter estimates from the two significant clusters (left and right RLPFC) (Fig. 2). In a whole-brain analysis within the lower-SES group, reasoning was positively related to cortical thickness in right cuneus and bilateral RLPFC (*p* <  .01, Fig. S4). No relationships between reasoning and whole-brain cortical thickness were observed in the higher-SES group.

**Fig. 3:**
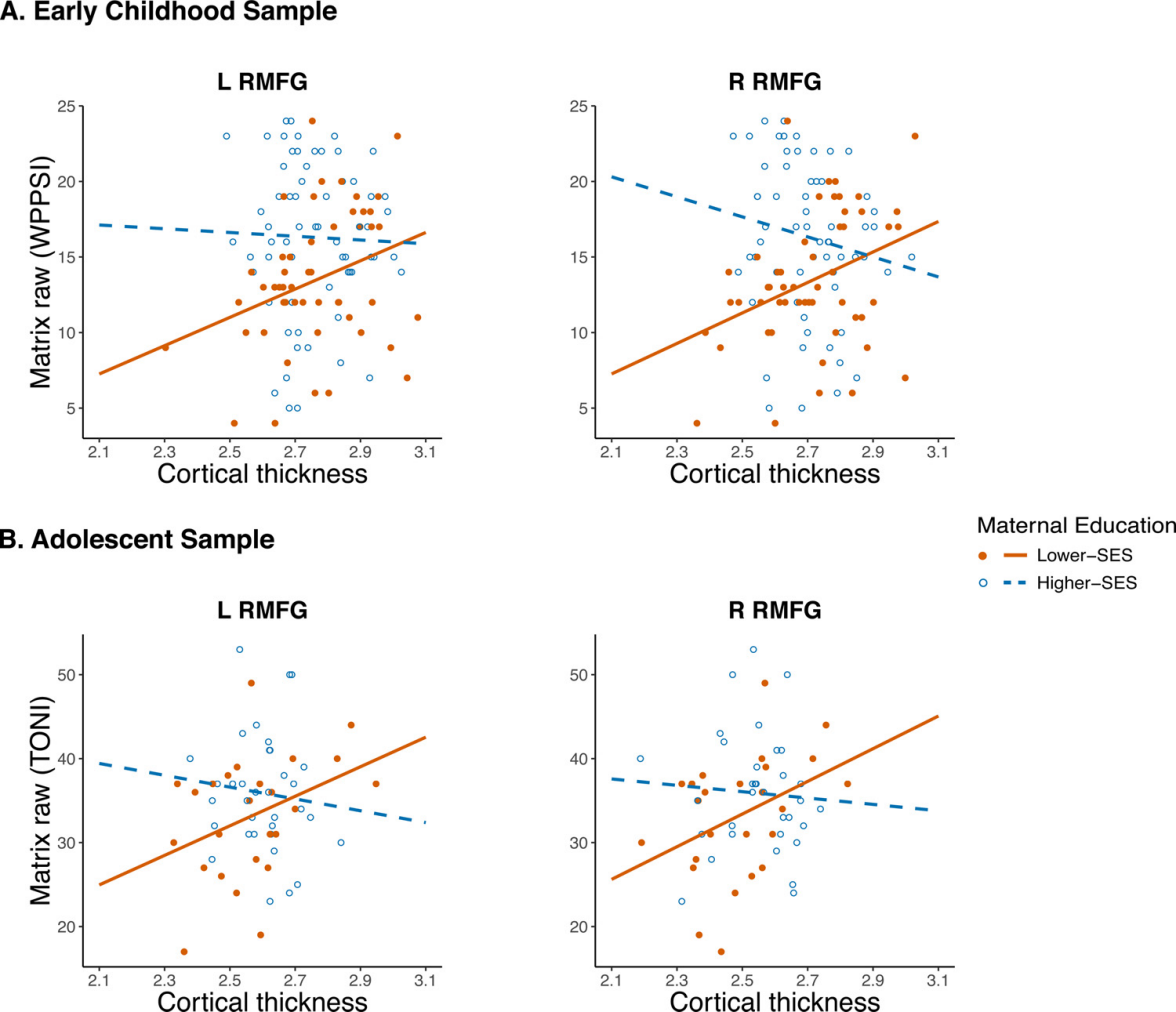
Relationships between matrix reasoning and RMFG ROI cortical thickness (mm) by SES group in A. the early childhood sample and B. adolescent sample. Age, gender, and image quality were included as covariates. Scatterplots show the relationship between each ROI’s thickness (defined from FreeSurfer aparc 2005 parcellations, adjusted for covariates) and reasoning by SES group (Lower-SES group, orange: maternal education < 16 years, Higher-SES group, blue: maternal education > = 16 years). In the early childhood sample, matrix reasoning was indexed by performance on the matrix reasoning subtest of the WPPSI. In the early childhood sample, matrix reasoning was indexed by performance on the matrix reasoning subtest of the Wechsler Preschool and Primary Scale of Intelligence (WPPSI). (For interpretation of the references to colour in this figure legend, the reader is referred to the web version of this article).

To better understand whether the interaction between matrix reasoning, maternal education and RLPFC thickness was driven by the higher- or lower-SES group, we ran statistics using the anatomically-defined bilateral rostral middle frontal gyrus (RMFG). The interaction between reasoning and maternal education was significant in left and right RMFG (L RMFG: b=- 2 × 10^−3^, 95% CI [- 4 × 10^−3^, - 1 × 10^−3^ ], t(107)=-2.67, p=.009; R RMFG: b=-3 x10^−3^, 95% CI [- 5 × 10^−3^, - 1 × 10^−3^ ], t(107)=-3.37, *p*=.001; Fig. 3A. The relationships between thickness in left and right RMFG and matrix reasoning were positive and significant only in the lower-SES group (L RMFG: b=0.01, 95% CI [ 1 × 10^−3^, 0.03], t(46)=2.19, p=.034; R RMFG: b=0.02, 95% CI [0.01, 0.03], t(46)=2.95, p=.005). In the higher-SES group, relationships between left and right RMFG thickness and matrix reasoning were not significant (L RMFG:  b= 2 × 10^−3^, 95% CI [-0.01, 0.01], t(57)=0.35, p=.725; R RMFG: b=- 5 × 10^−3^, 95% CI [-0.01, 4 × 10^−3^ ], t(57)=-1.13, p=.262).

**Fig. 4:**
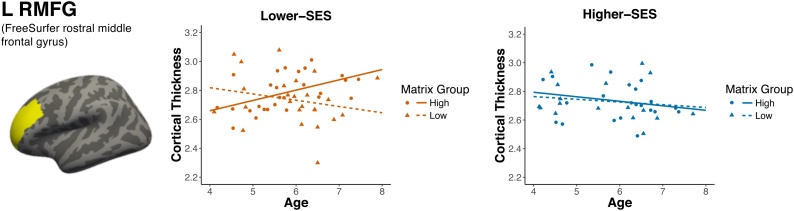
Three-way interaction between age, maternal education, and matrix reasoning on left rostral middle frontal gyrus (L RMFG) thickness (mm) in the early childhood sample. For plotting purposes only, children were split into two maternal education groups based on whether their mothers completed 4-year college (Lower-SES group, orange: maternal education < 16 years, Higher-SES group, blue: maternal education > = 16 years). For plotting purposes only, children were split into high (solid line) and low (dashed line) matrix reasoning groups based on the median matrix reasoning standard score within each SES group (high = circle, low = triangle). (For interpretation of the references to colour in this figure legend, the reader is referred to the web version of this article).

To test whether relationships between RLPFC thickness and age differed by SES and reasoning ability, we ran age X maternal education X matrix reasoning interactions on cortical thickness in anatomically-defined bilateral rostral middle frontal gyrus (RMFG). The 3-way interaction was significant for left RMFG (Table 1, Fig. 4). Specifically, children from lower-SES backgrounds with high reasoning ability showed a significant and positive relationship between left RMFG thickness and age (b=0.08, 95% CI [0.03, 0.14], t(22)=3.28, p=.003), while those with low reasoning ability showed no relationship (b=-0.03, 95% CI [-0.13, 0.06], t(20)=-0.70, p=.491). Children from higher-SES backgrounds showed no relationships between left RMFG thickness and age in either reasoning group (high reasoning: b=-0.02, 95% CI [-0.06, 0.03], t(30)=-0.90, p=.376; low reasoning: b=-0.01, 95% CI [-0.07, 0.05], t(22)=-0.25, p=.801). There were no main effects of age, maternal education, or matrix reasoning with left or right RMFG when evaluated in separate models (all *p* >  .3).

**Table 1:** Three-way interaction of age, maternal education, and matrix reasoning on the thickness of left and right rostral middle frontal gyrus (RMFG) in the early childhood sample.

Parameter	Estimate	SE	*t*	*p*
*L RMFG*				
Intercept	5.59	1.53	3.66	< .001
Mother Education	−0.18	0.09	−1.89	.062
Matrix Raw	−0.20	0.11	−1.75	.084
Age	−0.61	0.27	−2.22	.029*
Sex	0.04	0.03	1.48	.143
Image Quality	−0.00	0.01	−0.06	.950
Study	−0.05	0.03	−1.83	.070
Mother Education x Matrix Raw	0.01	0.01	1.87	.064
Mother Education x Age	0.04	0.02	2.20	.030*
Matrix Raw x Age	0.04	0.02	2.22	.029*
Mother Education x Matrix Raw x Age	−0.00	0.00	−2.30	.024*
*R RMFG*				
Intercept	4.58	1.53	2.99	.003
Mother Education	−0.12	0.10	−1.24	.217
Matrix Raw	−0.08	0.11	−0.73	.470
Age	−0.48	0.27	−1.76	.081
Sex	0.02	0.03	0.70	.486
Image Quality	−0.01	0.01	−0.47	.641
Study	−0.04	0.03	−1.48	.143
Mother Education x Matrix Raw	0.01	0.01	0.80	.424
Mother Education x Age	0.03	0.02	1.76	.081
Matrix Raw x Age	0.03	0.02	1.35	.179
Mother Education x Matrix Raw x Age	−0.00	0.00	−1.42	.160

L RMFG model: *F*(10, 104) = 2.28, *p* = .019, adj. *R*^2^ = 0.10. R RMFG model: *F*(10, 104) = 2.10, *p* = .031, adj. *R*^2^ = 0.09. Parameter estimates are unstandardized.

* p < .05

### 3.2 Adolescent sample

In the adolescent sample (n = 59), there was a positive trend between maternal education and matrix reasoning ($r\left(57\right)=\;.24$, p=.071; Fig. 1C). Splitting the sample into two groups based on maternal education showed high variability in scores within groups, and no significant group difference (t(57)=1.33, p=.189; Fig. 1D).

To extend the early childhood findings in this smaller sample, we focused our analyses on left and right RMFG (FreeSurfer aparc 2005). The interaction between reasoning scores and maternal education was not significant in the left or right RMFG in the adolescent sample (L RMFG: *b* =- 1 × 10^−3^, 95% CI [- 2 × 10^−3^, 4 × 10^−4^ ], t(52)=-1.33, p=.190; R RMFG: b=- 1 × 10^−3^, 95% CI [- 2 × 10^−3^, 1 × 10^−3^ ], $t\left(52\right)=-1.24$, p=.220). However, within adolescents from lower-SES backgrounds, greater cortical thickness in both left and right RMFG correlated with better reasoning scores (L RMFG: b=0.01, 95% CI [4 × 10^−4^, 0.02], t(20)=2.19, p=.040; R RMFG: b=0.01, 95% CI [ 1 × 10^−3^, 0.02], t(20)=2.37, p=.028; Fig. 3B). No significant relationships were found between RMFG thickness and reasoning scores in adolescents from higher-SES backgrounds (L RMFG: b=- 4 × 10^−4^, 95% CI [-0.01, 5 × 10^−3^ ], t(29)=-0.15, p=.883; R RMFG: b=- 4 × 10^−4^, 95% CI [-0.01, 0.01], t(29)=-0.12, p=.907). There were no main effects of age or matrix reasoning (p > .2) on RMFG cortical thickness, but there was a trend for maternal education(L RMFG: b=0.01, 95% CI [- 2 × 10^−3^, 0.02], t(55)=1.66, p=.103; R RMFG: b=0.01, 95% CI [- 2 × 10^−3^, 0.02], t(55)=1.61, p=.113). Three-way interactions amongst age, maternal education, and matrix reasoning were not significant (Table S2). We also ran a whole-brain interaction between maternal education and matrix reasoning on cortical thickness and found no significant clusters, even at the lenient threshold of cluster-wise *p* <  .05, cluster-forming threshold *p* <  .05.

## 4 Discussion

The relationship between RLPFC thickness and fluid reasoning differed by SES in children and adolescents. In young children from lower-SES backgrounds, but not from higher-SES backgrounds, thicker RLPFC was related to better reasoning ability. This pattern of results was also present in an independent sample of adolescents, although the full interaction was not significant. In the early childhood sample, we found that children from lower-SES backgrounds with higher reasoning ability showed a positive relationship between left RLPFC thickness and age, while those with lower reasoning ability showed a non-significant negative relationship. No interaction between age, reasoning ability, and cortical thickness was found in the higher-SES group. Thus, high achieving children from lower-SES backgrounds may have a unique relationship between RLPFC thickness and age in early childhood. Consistent with the evolutionary-developmental literature showing that individuals adapt to fit their environment, the current work suggests that the neural correlates of successful reasoning differ by early life environment (Ellis et al., 2017).

The location of the interaction between matrix reasoning and SES in RLPFC is consistent with evidence that this area is important for fluid reasoning, especially relational integration (Bunge et al., 2009; Christoff et al., 2001; Crone et al., 2009; Wendelken et al., 2011, 2012, 2016). Greater cortical thickness in this region could reflect increased local neural computational resources, an interpretation that would align with the finding that thicker RLPFC is also related to higher IQ later in development and adulthood (Choi et al., 2008; Karama et al., 2009; Menary et al., 2013; Narr et al., 2007). It is unclear why we do not observe relationships between cortical thickness and reasoning in individuals from higher-SES backgrounds in either age group. It is possible that these relationships emerge after age 16, or that there is greater diversity in how the brain supports reasoning in the higher-SES group. Furthermore, we found no significant clusters in the reasoning by maternal education whole-brain cortical thickness analysis in the adolescent sample. This could be due to decreased power in the smaller adolescent sample size and/or a weaker effect in this older sample.

Our results are consistent with other studies showing differences in brain-behavior relationships by SES, across functional and structural imaging modalities. Variability in arithmetic performance in children from lower-SES backgrounds was linked to activation of visuospatial areas, while variability in children from higher-SES backgrounds was linked to activation of verbal areas (Demir et al., 2015). During a reading task, phonological awareness correlated with greater activation of fusiform cortex in children from lower-, but not higher-, SES backgrounds (Noble et al., 2006). Adolescents from higher-SES backgrounds exhibited greater positive relationships between parietal activation on a working memory task and standardized math test scores than adolescents from lower-SES backgrounds (Finn et al., 2016). SES has also been shown to moderate relationships between brain structure and cognition: among children with thicker cortices, SES was more predictive of executive function, and less predictive of language skills (Brito et al., 2017). Other research has also shown that SES impacts relationships between brain structure and age (LeWinn et al., 2017). The present study goes beyond past research by examining how brain-behavior relationships differ by SES by specifically focusing on fluid reasoning at multiple stages of development.

Children with strong reasoning skills from lower-SES backgrounds were the only group to show a significant positive relationship between RLPFC thickness and age. All other groups of participants showed nonsignificant negative relationships between RLPFC thickness and age. However, the positive relationship between RLPFC thickness and age in high-performing children from lower-SES backgrounds was not significant in the adolescent sample, indicating that this relationship may be specific to early childhood development. While we do not know why differential early RLPFC development specifically benefits children from lower-SES backgrounds, one possibility is that slower development (thickening instead of thinning) is beneficial because it allows the brain to be more flexible and responsive to learning (Shaw et al., 2006; Yeatman et al., 2012). Another possibility is that increased neural resources are required to reason well in a challenging environment, so as other children are beginning to show effects of pruning, children from lower-SES environments with strong reasoning skills continue to show growth of RLPFC during early childhood.

Main effects of reasoning, SES, and age were limited to occipital cortex in the early childhood sample. We observed a positive relationship between matrix reasoning and cortical thickness only in lateral occipital cortex, in contrast to previous studies linking FSIQ to cortical thickness in older and broader age ranges (Choi et al., 2008; Karama et al., 2009; Menary et al., 2013; Narr et al., 2007). We saw a negative relationship between age and thickness of pericalcarine cortex, which contains primary visual cortex, consistent with multiple studies that show cortical thinning in sensory areas first, followed by thinning in association regions (Gogtay et al., 2004; Shaw et al., 2006; Sowell et al., 2004; Walhovd et al., 2016). We only found an effect of SES on cortical thickness at a lenient cluster-forming threshold in lateral occipital cortex, consistent with small cortical thickness differences by SES in early childhood (Piccolo et al., 2016) (note: SES-related volume differences have been observed in infancy, but may be driven by surface area rather than thickness (Betancourt et al., 2016; Hanson et al., 2013)). It is unclear why main effects clustered in occipital cortex, but it could be that relations between SES and cognition emerge as development progresses: in early childhood, these relationships are apparent in early-developing sensory regions, but the cortical footprint of such relationships spread throughout development.

This study has several limitations. First, it was cross-sectional, precluding direct evidence about relationships between age-related changes in cortical thickness and cognitive development. Second, given the observational nature of the study, we cannot determine whether environmental experiences associated with SES cause differential relationships between cortical thickness and reasoning. Third, this study relied on maternal education and income as proxy measures of SES. Fourth, this study included only one measure of fluid reasoning, so it is unclear whether our findings would generalize across multiple measures of reasoning, or across cognitive measures more broadly. Furthermore, given that this study focused on structural data, we cannot investigate the functional consequences of cortical thickness, nor can we confirm that the region of RLPFC that showed a relationship between thickness and reasoning in children from lower-SES backgrounds overlaps with functional regions that support reasoning. Finally, the study did not include other cognitive measures that were consistent across samples, or measures of early academic skills, so we could not test whether differential relationships between cortical thickness and reasoning are specific to reasoning, or whether they are related to, or predictive of, FSIQ or performance in school. Future longitudinal research with a wider set of environmental, cognitive, and academic measures is necessary to more fully understand whether optimal structural brain development differs by environment.

Understanding how neural correlates of cognition differ by childhood environment could inform the use of neural markers in intervention evaluation. Many interventions that work to narrow the income-achievement gap take years for effects to present (*e.g.*, Perry Preschool Project (Schweinhart and Barnes, 2005), Abecedarian Project (Ramey et al., 2000)) and subsequently years to evaluate. Neuroimaging could be used as a “checkpoint” or surrogate endpoint to provide more immediate feedback on the effectiveness of an intervention, as changes can appear in the brain prior to changes in behavior (Dumontheil and Klingberg, 2012; Gabrieli et al., 2015; Hoeft et al., 2007, 2011; Supekar et al., 2013; Ullman et al., 2014). However, most work on positive brain development has focused on children from higher-SES backgrounds, and, as we have demonstrated here, the principles of positive brain development may differ across SES environments. Thus, it is crucial to understand how positive brain development occurs differentially across varied SES experiences so as to promote brain development in a way that is valid for each child according to his or her environment.

## Data and code availability

All analysis scripts and demographic, behavioral, and ROI data are available through OSF (https://osf.io/unsy8/). Uncorrected whole brain surfaces for the main analysis looking at the relationship between cortical thickness and matrix reasoning by maternal education in the early childhood sample are also available through OSF.

## Declaration of interest

None to report.
