# Supplementary material for: Associations between cortical thickness and reasoning differ by socioeconomic status in development
Source: Dev Cogn Neurosci. 2019 Mar 23;36:100641. doi: 10.1016/j.dcn.2019.100641 (PMC6969225; doi:10.1016/j.dcn.2019.100641)
Supplement: Supplementary file 1 [file mmc1.docx]

**Supplemental Materials**

Associations between cortical thickness and reasoning differ by

socioeconomic status in development

**Analyses controlling for race and ethnicity**

We ran all main significant analyses with the RMFG ROI from FreeSurfer controlling for race (white = 1 or not white = 0) and ethnicity (Hispanic/Latino = 1 or not Hispanic/Latino = 0). In the ECS, the interaction between reasoning and maternal education was significant in the right and left RMFG when controlling for race (L RMFG: $b=-$2x10^-3^, 95% CI $[-$4x10^-3^, $-$5x10^-4^], $t(103)=-2.50$, $p=.014$ ; R RMFG: $b=-3$x10^-3^, 95% CI $[-$5x10^-3^, $-$1x10^-3^$]$, $t(103)=-3.27$, $p=.001$) and ethnicity (L RMFG: $b= -$2x10^-3^, 95% CI $[-$4x10^-3^, $-$4 x10^-4^], $t(103)=-2.44$, $p=.016$; R RMFG: $b=-$3x10^-3^, 95% CI$[-$5x10^-3^, $-$1x10^-3^$]$, $t(103)=-3.19$, $p=.002$). The relationship between thickness in the right RMFG and matrix reasoning was also positive and significant in the ECS lower-SES group when controlling for race and ethnicity (race: $b=$ 1.6x10^-2^, 95% CI $[$5x10^-3^, 2.8x10^-2^$]$, $t(42)=2.80$, $p=.008$, ethnicity: $b=$1.5x10^-2^, 95% CI $[$3x10^-3^, 2.6x10^-2^$]$, $t(42)=2.55$, $p=.014$) and trending for the left RMFG (race: $b=$1.2x10^-2^, 95% CI $[-$1x10^-3^, 2.4x10^-2^$]$, $t(42)=1.88$, $p=.066$, ethnicity: $b=0.01$, 95% CI $[-$1x10^-3^, 2.4x10^-2^$]$, $t(42)=1.85$, $p=.071$). Furthermore, the age X maternal education X matrix reasoning interactions on cortical thickness were significant for left RMFG when controlling for race ($b=-3$x10^-3^, 95% CI $[-$5x10^-3^, $-$5x10^-4^], $t(100)=-2.42$, $p=.017$) and ethnicity ($b=-3$x10^-3^, 95% CI $[-0.01$, $-$5x10^-4^], $t(100)=-2.38$, $p=.019$). Finally, in the AS, the relationship between left and right RMFG and reasoning was still significant in the lower-SES group when controlling for race (L RMFG: $b=9$x10^-3^, 95% CI [3x10^-4^, $0.02]$, $t(19)=2.17$, $p=.043$, R RMFG: $b=9$x10^-3^, 95% CI $[1$x10^-3^, $0.02]$, $t(19)=2.29$, $p=.033$) and ethnicity only in the right RMFG (L RMFG: $b=0.01$, 95% CI $[-2$x10^-3^, $0.02]$, $t(18)=1.72$, $p=.103$, R RMFG: $b=0.01$, 95% CI $[1$x10^-3^, $0.02]$, $t(18)=2.37$, $p=.029$).

**Income Analyses**

Income positively correlated with matrix reasoning scores ($r(112)=.26$, $p=.005$; Figure S5). Similarly, when we split the sample into two groups based on median family income in the greater Boston area in 2016 (< 80K/year or >= 80K/year), there was a significant difference in matrix reasoning by income group ($t(112)=3.31$, $p=.001$). There were no main effects of income on cortical thickness at the cluster-forming threshold of *p* < .005 (for more lenient thresholds, see Figure S6).

There was a significant interaction between income and matrix reasoning on whole-brain cortical thickness in lateral occipital cortex and RLPFC at the lower cluster-forming threshold of *p* <.05 (Figure S7 for all thresholds). To understand the direction of the interaction, and to confirm that it was not driven by outliers, we plotted parameter estimates from the lateral occipital cortex cluster (Figure S7). Within the lower-income group, reasoning was positively related to whole-brain cortical thickness in bilateral RLPFC (clustering forming threshold *p* <.05) and the right cuneus (clustering forming threshold *p* < .01, Figure S8). No relationships between reasoning and whole-brain cortical thickness were observed in the higher-income group.

We also tested whether there was a significant income by matrix reasoning interaction with the anatomically defined RMFG (FreeSurfer aparc 2005). The interaction between reasoning and income was significant in the right RMFG and trending in the left RMFG (L RMFG: $b=-$6x10^-5^, 95% CI $[-$1.3x10^-4^, 1x10^-5^$]$, $t(107)=-1.66$, $p=.100$; R RMFG: $b=-$8x10^-5^, 95% CI $[-$1.5x10^-4^, 1x10^-5^$]$, $t(107)=-2.35$, $p=.020$). Within children from lower-income backgrounds, greater cortical thickness in both left and right RMFG correlated with better reasoning scores (L RMFG: $b=0.01$, 95% CI $[2$x10^-3^, $0.02]$, $t(52)=2.41$, $p=.019$; R RMFG: $b=0.01$, 95% CI $[2$x10^-3^, $0.02]$, $t(52)=2.45$, $p=.018$). No significant relationships were found between RMFG thickness and reasoning scores in children from higher-income backgrounds (L RMFG: $b=-2$x10^-3^, 95% CI $[-0.01$, $0.01]$, $t(51)=-0.45$, $p=.651$; R RMFG: $b=-0.01$, 95% CI $[-0.02$, $2$x10^-3^$]$, $t(51)=-1.52$, $p=.134$).

We also tested whether relationships between RLPFC thickness and age differed by SES (when operationalized as income) and reasoning ability. We ran age X income X matrix reasoning interactions on cortical thickness in anatomically-defined bilateral RMFG. The 3-way interaction was significant for the left and right RMFG (Table S3, Figure S9). Children from lower-income backgrounds with high reasoning ability showed a significant and positive relationship between left RMFG thickness, but not right RMFG thickness, and age (L RMFG: $b=0.08$, 95% CI $[0.02$, $0.13]$, $t(28)=2.93$, $p=.007$; R RMFG: $b=0.05$, 95% CI $[-0.02$, $0.12]$, $t(28)=1.51$, $p=.141$), while those with low reasoning ability showed no significant relationships between RMFG and age (L RMFG: $b=-0.06$, 95% CI $[-0.14$, $0.02]$, $t(20)=-1.47$, $p=.157$; R RMFG: $b=-0.06$, 95% CI $[-0.14$, $0.02]$, $t(20)=-1.47$, $p=.158$). Children from higher-SES backgrounds showed no significant relationships between right and left RMFG thickness and age in either reasoning group (high reasoning L RMFG: $b=-0.03$, 95% CI $[-0.08$, $0.01]$, $t(25)=-1.49$, $p=.148$; high reasoning R RMFG: $b=-0.02$, 95% CI $[-0.07$, $0.03]$, $t(25)=-0.87$, $p=.394$; low reasoning L RMFG:$b=-0.004$, 95% CI $[-0.08$, $0.07]$, $t(21)=-0.11$, $p=.912$; low reasoning R RMFG:$b=3$x10^-3^, 95% CI $[-0.05$, $0.06]$, $t(21)=0.10$, $p=.923$). There was no main effect of income with left or right RMFG, controlling for sex, study, and T1 image quality (L RMFG: $b=$1x10^-5^, 95% CI [3.5x10^-^4, 3.6x10^-4^], $t(110)=0.03$, $p=.973$ , R RMFG: $b=$2x10^-4^, 95% CI [2x10^-4^, 5x10^-4^], $t(110)=0.88$, $p=.382$).

|  | | **Full Sample**  (*ECS* n = 115)  (*AS* n = 59) | | **Lower-SES**  (*ECS* n = 52)  (*AS* n = 25) | | **Higher-SES**  (*ECS* n = 63)  (*AS* n = 34) | |
| --- | --- | --- | --- | --- | --- | --- | --- |
|  |  | **Mean** | **SD** | **Mean** | **SD** | **Mean** | **SD** |
| *ECS* |  |  |  |  |  |  |  |
|  | Age | 5.85 | 0.96 | 5.84 | 0.88 | 5.87 | 1.03 |
|  | Matrix Reasoning (SS) | 10.80 | 2.85 | 9.60 | 2.71 | 11.81 | 2.59 |
|  | Maternal Education | 15.47 | 2.91 | 12.71 | 1.29 | 17.75 | 1.59 |
|  | Income (in thousands) | 93.89 | 72.96 | 44.32 | 31.68 | 134.80 | 72.12 |
|  | T1 quality rating | -0.002 | 0.98 | 0.09 | 1.01 | -0.08 | 0.97 |
|  | L RMFG thickness (mm) | 2.76 | 0.14 | 2.76 | 0.15 | 2.76 | 0.13 |
|  | R RMFG thickness (mm) | 2.71 | 0.14 | 2.71 | 0.16 | 2.71 | 0.12 |
| *AS* |  |  |  |  |  |  |  |
|  | Age | 14.44 | 0.55 | 14.52 | 0.60 | 14.37 | 0.51 |
|  | Matrix Reasoning (SS) | 98.39 | 9.73 | 96.44 | 9.47 | 99.82 | 9.81 |
|  | Maternal Education | 15.42 | 3.24 | 12.40 | 2.25 | 17.65 | 1.65 |
|  | T1 quality rating | 1.99 | 0.35 | 1.92 | 0.37 | 2.05 | 0.33 |
|  | L RMFG thickness (mm) | 2.59 | 0.13 | 2.57 | 0.15 | 2.61 | 0.10 |
|  | R RMFG thickness (mm) | 2.52 | 0.14 | 2.48 | 0.15 | 2.55 | 0.13 |

**Table S1. Demographics from the Early Childhood Sample (ECS) and the Adolescent Sample (AS), further broken down by SES (Lower-SES group: maternal education < 16 years, Higher-SES group: maternal education >= 16 years).** In the ECS, matrix reasoning was indexed by performance on the matrix reasoning subtest of the WPPSI (Standard score mean = 10, SD = 3). In the AS, matrix reasoning was indexed by performance on the TONI (Standard score mean = 100, SD = 15).

**Figure S1. Visual inspection instructions for structural image quality rating.** Trained coders were told to visually inspect gray and white matter boundaries and medial temporal lobe and orbitofrontal dropout. Additionally, coders looked for motion artifacts such as ringing. T1 image quality was rated on a scale of 1 (highest quality) to 4 (lowest quality) based on the above guide.

**Figure S2. Main effects of maternal education, matrix reasoning, and age on cortical thickness in the early childhood sample.** Age, gender, image quality, and study were included as covariates in all models (age was not a covariate in the model of age). Results were cluster corrected for multiple comparisons using Monte Carlo simulations and are shown here at cluster-forming *p* < .05, .01, and .005 (cluster-wise *p* < .05, adjusted for both hemispheres).

**Figure S3. Interaction between matrix reasoning and maternal education with cortical thickness at three different cluster-forming thresholds in the early childhood sample.** Age, gender, image quality, and study were included as covariates. Results were cluster corrected for multiple comparisons using Monte Carlo simulations (cluster-forming *p* < .05, .01, and .005, cluster-wise *p* < .05, adjusted for both hemispheres).

**Figure S4. Matrix reasoning and cortical thickness in the lower-SES group in the early childhood sample.** Age, gender, image quality, maternal education, and study were included as covariates. Results were cluster corrected for multiple comparisons using Monte Carlo simulations (cluster-forming *p* < .05, .01, and .005, cluster-wise *p* < .05, adjusted for both hemispheres). Results were non-significant in the higher-SES group.

| **Parameter** | **Estimate** | **SE** | ***t*** | ***p*** |
| --- | --- | --- | --- | --- |
| *L RMFG* |  |  |  |  |
| Intercept | -6.20 | 11.92 | -0.52 | .605 |
| Matrix Raw | 0.18 | 0.36 | 0.50 | .621 |
| Mother Education | 0.78 | 0.76 | 1.02 | .311 |
| Age | 0.56 | 0.82 | 0.68 | .502 |
| sex | 0.05 | 0.04 | 1.54 | .131 |
| Image Quality | 0.02 | 0.05 | 0.33 | .743 |
| Matrix Raw x Mother Education | -0.02 | 0.02 | -0.77 | .447 |
| Matrix Raw x Age | -0.01 | 0.02 | -0.45 | .653 |
| Mother Education x Age | -0.05 | 0.05 | -0.98 | .333 |
| Matrix Raw x Mother Education x Age | 0.00 | 0.00 | 0.73 | .469 |
| *R RMFG* |  |  |  |  |
| Intercept | 1.88 | 12.77 | 0.15 | .884 |
| Matrix Raw | -0.07 | 0.39 | -0.17 | .862 |
| Mother Education | -0.12 | 0.81 | -0.15 | .885 |
| Age | -0.01 | 0.88 | -0.01 | .991 |
| Sex | 0.08 | 0.04 | 2.05 | .045* |
| Image Quality | 0.04 | 0.06 | 0.65 | .517 |
| Matrix Raw x Mother Education | 0.01 | 0.02 | 0.40 | .693 |
| Matrix Raw x Age | 0.01 | 0.03 | 0.21 | .831 |
| Mother Education x Age | 0.01 | 0.06 | 0.19 | .848 |
| Matrix Raw x Mother Education x Age | -0.00 | 0.00 | -0.43 | .667 |

**Table S2. Three-way interaction of age, maternal education, and matrix reasoning on thickness of left and right rostral middle frontal gyrus (RMFG) in the adolescent sample.** L RMFG model: *F*(9, 49) = 1.40, *p* =.205, adj. *R*^2^ = 0.06. R RMFG model: *F*(9, 49) = 1.80, *p* =.092, adj. *R*^2^ = 0.11. Parameter estimates are unstandardized. * *p* < .05

**Figure S5.** **Income correlated with Matrix Reasoning standard score (SS) in the early childhood sample.** A. Income correlated with matrix reasoning age-normed standard score (Matrix SS) in the early childhood sample (*r*(112) = .26, *p* = .005). B. Matrix SS differed by Income group in the early childhood sample (Lower-income: annual income < 80K, Higher-income: annual income >= 80K) (*t*(112) = 3.31, *p* = .001).

**Figure S6. Main effect of income on cortical thickness in the early childhood sample.** Age, gender, image quality, and study were included as covariates. Results were cluster corrected for multiple comparisons using Monte Carlo simulations and are shown here at cluster-forming *p* < .05 (results were not significant at cluster-forming *p* <.01; cluster-wise *p* < .05, adjusted for both hemispheres).

**Figure S7. Interaction between matrix reasoning and income with cortical thickness at three different cluster-forming thresholds in the early childhood sample.** Age, gender, image quality, and study were included as covariates. Results were cluster corrected for multiple comparisons using Monte Carlo simulations (cluster-forming *p* < .05, .01, and .005, cluster-wise *p* < .05, adjusted for both hemispheres). The scatterplot shows interaction result with extracted parameter estimates (adjusted for covariates) in right lateral occipital cortex (in mm). Matrix reasoning was measured with the *Wechsler Preschool and Primary Scale of Intelligence (*WPPSI). Income is plotted as a binary variable for display purposes only (Lower-income group, orange: annual income < 80K, Higher-income group, blue: annual income >= 80K).

**Figure S8. Matrix reasoning and cortical thickness in the lower-income group in the early childhood sample.** Age, gender, image quality, maternal education, and study were included as covariates. Results were cluster corrected for multiple comparisons using Monte Carlo simulations (cluster-forming *p* < .05, .01, and .005, cluster-wise *p* < .05, adjusted for both hemispheres). Results were non-significant in the higher-income group.

| **Parameter** | **Estimate** | **SE** | ***t*** | ***p*** |
| --- | --- | --- | --- | --- |
| *L RMFG* |  |  |  |  |
| Intercept | 3.63 | 0.42 | 8.62 | < .001 |
| Income | -0.01 | 0.00 | -2.33 | .022* |
| Matrix Raw | -0.05 | 0.03 | -1.73 | .087 |
| Age | -0.18 | 0.08 | -2.36 | .020* |
| Sex | 0.02 | 0.03 | 0.82 | .417 |
| Image Quality | 0.00 | 0.01 | 0.27 | .784 |
| Study | -0.04 | 0.03 | -1.50 | .137 |
| Income x Matrix Raw | 0.00 | 0.00 | 2.31 | .023* |
| Income x Age | 0.00 | 0.00 | 2.57 | .012* |
| Matrix Raw x Age | 0.01 | 0.01 | 2.15 | .034* |
| Income x Matrix Raw x Age | -0.00 | 0.00 | -2.67 | .009* |
| *R RMFG* |  |  |  |  |
| Intercept | 3.43 | 0.42 | 8.08 | < .001 |
| Income | -0.01 | 0.00 | -1.83 | .070 |
| Matrix Raw | -0.04 | 0.03 | -1.18 | .239 |
| Age | -0.16 | 0.08 | -2.09 | .039* |
| Sex | 0.01 | 0.03 | 0.29 | .774 |
| Image Quality | -0.01 | 0.01 | -0.42 | .676 |
| Study | -0.03 | 0.03 | -1.01 | .314 |
| Income x Matrix Raw | 0.00 | 0.00 | 1.49 | .139 |
| Income x Age | 0.00 | 0.00 | 2.33 | .022* |
| Matrix Raw x Age | 0.01 | 0.01 | 1.65 | .101 |
| Income x Matrix Raw x Age | -0.00 | 0.00 | -2.04 | .044* |
|  |  |  |  |  |

**Table S3. Three-way interaction of age, income, and matrix reasoning on thickness of left and right rostral middle frontal gyrus (RMFG) in the early childhood sample.** L RMFG model: *F*(10, 104) = 1.902, *p* =.052, adj. *R*^2^ = 0.07. R RMFG model: *F*(10, 104) = 1.614, *p* =.113, adj. *R*^2^ = 0.05. Parameter estimates are unstandardized. * *p* < .05

**Figure S9.** **Three-way interaction between age, income, and matrix reasoning on left and right rostral middle frontal gyrus (RMFG) thickness (mm) in the early childhood sample**. For plotting purposes only, children were split into two income groups (Lower-income group, orange: annual income < 80K, Higher-income group, blue: annual income >= 80K). For plotting purposes only, children were split into high (solid line) and low (dashed line) matrix reasoning groups based on the median matrix reasoning standard score within each SES group (high = circle, low = triangle).
